# Regulatory T cells in pregnancy disorders: a multi-dimensional framework for biomarkers and therapeutic strategies

**DOI:** 10.3389/fimmu.2026.1850984

**Published:** 2026-07-01

**Authors:** Ning Zhang, Jun Zhou, Wenxue Ma, Jing Li

**Affiliations:** 1 Department of Obstetrics, The Affiliated Hospital of Qingdao University, Qingdao, Shandong, China; 2 Sanford Stem Cell Institute, Department of Medicine, and Moores Cancer Center, University of California San Diego, La Jolla, CA, United States; 3 Shandong Provincial Maternal and Child Health Care Hospital Affiliated to Qingdao University, Qingdao, Shandong, China; 4 Key Laboratory of Maternal & Fetal Medicine of National Health Commission of China, Qingdao, Shandong, China

**Keywords:** immune biomarkers, immune tolerance, maternal-fetal interface, pregnancy disorders, regulatory T cells

## Abstract

Pregnancy represents a unique immunological state in which the maternal immune system must maintain tolerance toward the semi-allogeneic fetus while preserving protective immunity against pathogens. Regulatory T cells (Tregs) are central to this balance, coordinating immune suppression, tissue remodeling, and vascular adaptation throughout gestation. Increasing evidence implicates Treg dysregulation in pregnancy disorders, including recurrent pregnancy loss, preeclampsia, and spontaneous preterm labor. Notably, pathological alterations extend beyond changes in cell abundance and involve multi-dimensional defects in suppressive function, lineage stability, spatial distribution, and regulatory signaling. Advances in high-dimensional immune profiling, including single-cell, spatial, and multi-omics approaches have revealed substantial heterogeneity in Treg populations at the maternal-fetal interface and enabled identification of immune signatures with diagnostic and prognostic potential. These insights are reshaping biomarker development from static measurements toward functionally and spatially resolved immune profiling. In this review, we propose a stage-specific and multi-dimensional framework for understanding Treg biology in pregnancy and systematically compare Treg alterations across major pregnancy disorders. We further evaluate the translational potential of circulating and decidual Treg signatures as biomarkers and discuss emerging therapeutic strategies aimed at restoring immune tolerance. Taken together, this framework positions Treg biology as a foundation for biomarker-guided diagnosis and mechanism-based therapeutic interventions in pregnancy complications.

## Highlights

- Tregs coordinate immune tolerance and tissue remodeling at the maternal-fetal interface.
- Treg dysfunction involves multi-dimensional defects beyond changes in cell abundance.
- Single-cell and spatial technologies reveal functional and spatial heterogeneity of Tregs.
- Circulating and decidual Tregs provide complementary biomarker information.
- Targeting Treg pathways offers mechanism-based strategies to restore immune tolerance.

## 1 Introduction

Pregnancy represents one of the most remarkable immunological adaptations in human biology. The developing fetus carries paternal antigens and is therefore recognized by the maternal immune system as a semi-allogeneic biological entity (1–3). Under normal circumstances, foreign antigens elicit immune rejection; however, during pregnancy, the maternal immune system establishes a finely tuned balance between immune tolerance and protective immunity that allows fetal development while preserving host defense against pathogens (4–6). This delicate equilibrium is orchestrated at the maternal-fetal interface, a highly specialized microenvironment composed of trophoblast cells, decidual stromal cells, and diverse immune populations including uterine natural killer cell (uNK) cells, macrophages, dendritic cells, and T lymphocytes (7, 8). Among these immune subsets, regulatory T cells (Tregs) have emerged as critical mediators of maternal-fetal immune tolerance (9, 10).

Tregs, defined by expression of the transcription factor Forkhead box P3 (FOXP3) and key immunosuppressive mediators such as cytotoxic T-lymphocyte-associated protein 4 (CTLA-4), IL-10, and transforming growth factor beta (TGF-β), are central to maintaining immune homeostasis (11, 12). Tregs expand systemically and within the decidua, where they restrain maternal immune activation and promote tolerance to fetal antigens (6, 13). Beyond immune suppression, Tregs actively regulate placental development by modulating uNK cell function, directing macrophage polarization, and facilitating trophoblast invasion and vascular remodeling (14, 15). These integrated roles position Tregs at the interface of immune regulation and tissue adaptation during gestation (14, 16).

Disruption of Treg-mediated immune regulation has been increasingly implicated in a wide range of pregnancy complications (17, 18). Reduced Treg abundance, impaired suppressive function, and imbalances between Tregs and pro-inflammatory T-cell subsets such as T helper type 1 (Th1) and T helper type 17 (Th17) cells have been reported in conditions including recurrent pregnancy loss, preeclampsia, spontaneous preterm labor (PTL), and implantation failure (19, 20). Despite growing recognition of the importance of Tregs in pregnancy immunology, the clinical translation of these findings remains limited (21, 22). Current diagnostic approaches for pregnancy disorders rely largely on clinical symptoms and nonspecific laboratory markers that often appear only after pathological processes have progressed (23, 24). Similarly, therapeutic strategies remain largely supportive rather than mechanism-based, highlighting the need for improved biomarkers and targeted immunomodulatory interventions (25, 26).

Although numerous reviews have summarized the immunological roles of Tregs in pregnancy, many of these studies remain largely descriptive and focus primarily on mechanistic pathways within specific disorders (27, 28). Relatively few analyses have systematically evaluated the translational potential of Treg-related immune signatures as diagnostic or predictive biomarkers or examined how mechanistic insights can inform targeted therapeutic strategies (12, 29, 30). Moreover, emerging advances in single-cell technologies, spatial immune profiling, and multi-omics approaches are rapidly reshaping our understanding of Treg heterogeneity and function at the maternal-fetal interface, yet these advances have not been fully integrated into clinically oriented or conceptually unified frameworks (31–33).

In this context, a critical gap remains in bridging mechanistic insights into Treg biology with clinically actionable applications (34). Specifically, it is unclear which dimensions of Treg dysregulation such as quantitative changes, functional impairment, lineage instability, or spatial redistribution are most relevant for disease prediction, stratification, and therapeutic targeting (35). Addressing this gap requires a conceptual framework that integrates Treg biology across multiple layers, from cellular function and tissue-specific dynamics to immune network interactions and biomarker development. Such an approach is essential for distinguishing descriptive immunological observations from clinically meaningful immune signatures.

To address this gap, we propose a multi-dimensional framework of Treg dysregulation encompassing four interconnected axes: quantitative abundance, functional competence, lineage stability, and spatial distribution within the maternal-fetal interface. We argue that these dimensions are differentially perturbed across pregnancy disorders and that their integration provides a more precise basis for biomarker development and therapeutic targeting than any single parameter alone. This framework enables a shift from descriptive immunophenotyping toward mechanism-based interpretation of immune dysfunction in pregnancy.

## 2 Treg biology in normal pregnancy: a stage-specific framework

Successful pregnancy requires dynamic immunological adaptations that evolve throughout gestation (6, 36). Rather than representing a uniform state of immune suppression, pregnancy involves stage-specific modulation of immune responses across distinct developmental phases, including preconception immune priming, peri-implantation immune adaptation (approximately days 6–10 post-fertilization), early placental development during the first trimester, maintenance of fetal tolerance throughout mid-gestation, and coordinated immune reactivation during late pregnancy and the postpartum period (37, 38). Within this framework, Tregs function as central regulators that integrate immune tolerance with tissue remodeling and developmental processes, coordinating context-dependent immune responses across gestation (39, 40).

### 2.1 Preconception immune priming

Immune tolerance during pregnancy is thought to begin before embryo implantation through early immune priming events (4, 41). Experimental murine studies indicate that exposure to paternal antigens in seminal fluid promotes the expansion of antigen-specific Tregs in the female reproductive tract and peripheral immune system (42–44). These Tregs may establish an immunological memory-like regulatory state that facilitates subsequent recognition and tolerance of fetal antigens during early pregnancy (1, 45).

In experimental animal models, seminal plasma provides a complex immunomodulatory milieu containing factors such as transforming growth factor-β (TGF-β), prostaglandins, and cytokines that promote Treg differentiation and expansion within uterine-draining lymph nodes (46, 47). This priming phase can therefore be viewed as a preparatory step that conditions the maternal immune system toward tolerance before direct maternal-fetal interaction occurs. This process likely occurs during the peri-conception period, although the precise timing, duration, and magnitude of such immune conditioning remain incompletely defined. Failure of this early programming may predispose to inadequate tolerance establishment during implantation, highlighting the potential importance of preconception immune conditioning in reproductive success (41, 48).

Human observational data provide indirect support for this concept. Epidemiological studies suggest that prolonged exposure to paternal seminal antigens, including longer duration of sexual cohabitation before conception and prior successful pregnancies with the same partner, may be associated with reduced risk of certain pregnancy complications such as preeclampsia, whereas partner change may attenuate this protective effect. Although these observations are consistent with adaptive immune conditioning, direct mechanistic evidence linking these effects specifically to Treg-mediated tolerance in humans remains limited.

### 2.2 Implantation and early immune tolerance

The implantation stage represents a critical immunological checkpoint in which maternal immune tolerance must be rapidly established in response to direct exposure to fetal alloantigens (40, 48, 49). In humans, implantation occurs within a relatively narrow peri-implantation window, making this one of the earliest critical periods for Treg-mediated immune adaptation. During this phase, Tregs accumulate in both the decidua and peripheral circulation, where they limit maternal effector T-cell responses and facilitate immune adaptation to the developing embryo (50, 51).

Mechanistically, Tregs exert their effects through coordinated cytokine-dependent and contact-mediated pathways, including CTLA-4 and PD-1 signaling, which suppress effector T-cell activation and stabilize local immune tolerance (12, 52). Beyond direct regulation of adaptive immunity, Tregs also shape innate immune responses at the maternal-fetal interface. Decidual immune populations including uNK cells, macrophages, and dendritic cells are functionally modulated through Treg-dependent signaling networks that constrain excessive inflammation while preserving the controlled immune activation required for implantation (8, 14, 53).

Conceptually, implantation can therefore be viewed as a transition from immune priming to active immune regulation, in which Tregs function as gatekeepers that balance inflammatory cues required for tissue invasion with tolerance mechanisms necessary for fetal acceptance. Disruption of this process, extravillous trophoblasts (EVTs) invade the maternal decidua and initiate spiral artery remodeling to establish adequate placental perfusion. This physiologic invasion occurs within a tightly regulated pro-inflammatory microenvironment characterized by coordinated cytokine, chemokine, and immune cell interactions that support tissue remodeling while preventing excessive inflammatory damage. Disruption of this balance may lead to implantation failure or early pregnancy loss, underscoring the critical role of Treg-mediated control during this stage.

### 2.3 Placental development and immune adaptation

Following implantation, placental development requires extensive remodeling of the uterine environment (54, 55). This phase is particularly prominent during the first trimester, when trophoblast invasion and spiral artery remodeling establish the structural and vascular foundation for ongoing fetal development. These processes position Tregs not merely as suppressive immune cells but as regulators of tissue adaptation, linking immune tolerance to vascular remodeling. During this stage, Tregs facilitate trophoblast invasion and spiral artery remodeling, processes essential for establishing adequate placental blood flow (14, 56). At the maternal-fetal interface, Tregs coordinate interactions among immune and non-immune cell populations to maintain immune equilibrium while supporting structural remodeling of placental tissues (40, 57).

One of the key functions of Tregs during placentation is the modulation of uNK cell activity (14, 58). Although uNK cells constitute the largest immune population within the decidua during early pregnancy, their activation state must be tightly regulated to preserve trophoblast integrity while maintaining their specialized roles in vascular remodeling and placental development (8, 59). Tregs contribute to this regulation through immunosuppressive cytokines such as IL-10 and TGF-β, as well as contact-dependent regulatory interactions that limit NK-cell activation, restrain excessive cytotoxicity, and promote a pro-angiogenic phenotype that supports vascular remodeling (12, 58, 60). In parallel, Tregs influence macrophage polarization toward anti-inflammatory M2-like phenotypes, further facilitating tissue repair and placental development (61, 62).

Collectively, this dual role of Tregs in immune regulation and tissue remodeling suggests that defects in Treg function may simultaneously disrupt immune homeostasis and placental architecture. Such coupling between immune imbalance and structural dysfunction provides a mechanistic link to pregnancy complications, particularly preeclampsia, where impaired vascular remodeling and heightened inflammation coexist.

### 2.4 Maintenance of fetal tolerance during gestation

As pregnancy progresses, the immunological challenge shifts from tolerance establishment to tolerance maintenance, requiring sustained and dynamically regulated Treg function (9, 63). This maintenance phase predominates during mid-gestation, when sustained immune tolerance becomes essential for continued fetal growth. During this period, both circulating and decidual Tregs remain elevated relative to non-pregnant conditions, reflecting a stable regulatory state that supports ongoing fetal development (27, 64). These Tregs preserve immune equilibrium by restraining maternal responses to fetal antigens while maintaining competence against infectious threats (65).

Mechanistically, this sustained regulatory state depends on reinforcement of Treg stability and function through hormonal and signaling inputs. Progesterone- and estrogen-mediated pathways, together with cytokine signaling networks, contribute to the maintenance of FOXP3 expression and suppressive capacity, thereby stabilizing the Treg lineage under physiological conditions (17, 66). This phase can therefore be conceptualized as a state of functional stabilization, in which Tregs resist inflammatory perturbation and maintain consistent regulatory activity.

Experimental animal studies suggest that Treg-mediated maternal tolerance may involve antigen-responsive or antigen-specific mechanisms directed toward paternal/fetal alloantigens (6, 13, 67). However, direct evidence demonstrating well-defined fetal alloantigen-specific Treg populations in humans remains limited. Thus, fetal tolerance in human pregnancy is more appropriately understood as a dynamically regulated process involving both selective immune adaptation and broader immunoregulatory control rather than exclusively generalized immune suppression (4, 68). Disruption of this balance may compromise both immune stability and fetal development, contributing to pregnancy complications.

Recent high-dimensional human studies further support the concept that Treg biology during pregnancy is highly dynamic rather than static. For example, a recent study identified evolving Treg heterogeneity and trajectory changes across human pregnancy, reinforcing the concept of stage-specific functional adaptation in maternal immune regulation (9). These human findings provide important translational support for temporal models of Treg-mediated maternal-fetal tolerance that have historically been informed largely by experimental animal studies.

### 2.5 Immune reset in late pregnancy and postpartum

Toward late gestation and following delivery, the maternal immune system undergoes a coordinated transition from a tolerogenic to a pro-inflammatory state, reflecting the resolution phase of pregnancy-associated immune adaptation (69, 70). This transition is most evident during late gestation, labor, and the early postpartum period, when controlled inflammatory activation supports parturition and immune restoration. However, immune re-equilibration is not immediate and may extend over weeks to months following delivery as pregnancy-associated endocrine signals progressively normalize (69). During this period, both the abundance and suppressive activity of Tregs decline, accompanied by a relative increase in pro-inflammatory immune responses (71, 72). This shift is functionally linked to parturition, where controlled inflammation contributes to uterine activation and tissue remodeling.

Conceptually, this phase represents an immune reset, in which the regulatory dominance established during earlier stages is progressively reversed. The reduction in Treg-mediated suppression permits reactivation of effector immune pathways, facilitating both delivery and postpartum immune restoration. However, the mechanisms governing the timing and coordination of this transition remain incompletely understood and likely involve interactions among hormonal withdrawal, inflammatory signaling, and tissue remodeling processes (37).

Importantly, dysregulation of this transition may have pathological consequences. Premature or excessive loss of Treg function may contribute to inflammatory-driven complications such as PTL, whereas delayed or dysregulated immune re-equilibration may delay restoration of normal maternal immune responsiveness and potentially increase susceptibility to postpartum infection or persistent inflammatory dysregulation (19). These considerations highlight that Treg biology is not only critical for maintaining pregnancy but also for ensuring appropriate resolution of the tolerogenic state.

**Figure 1:**
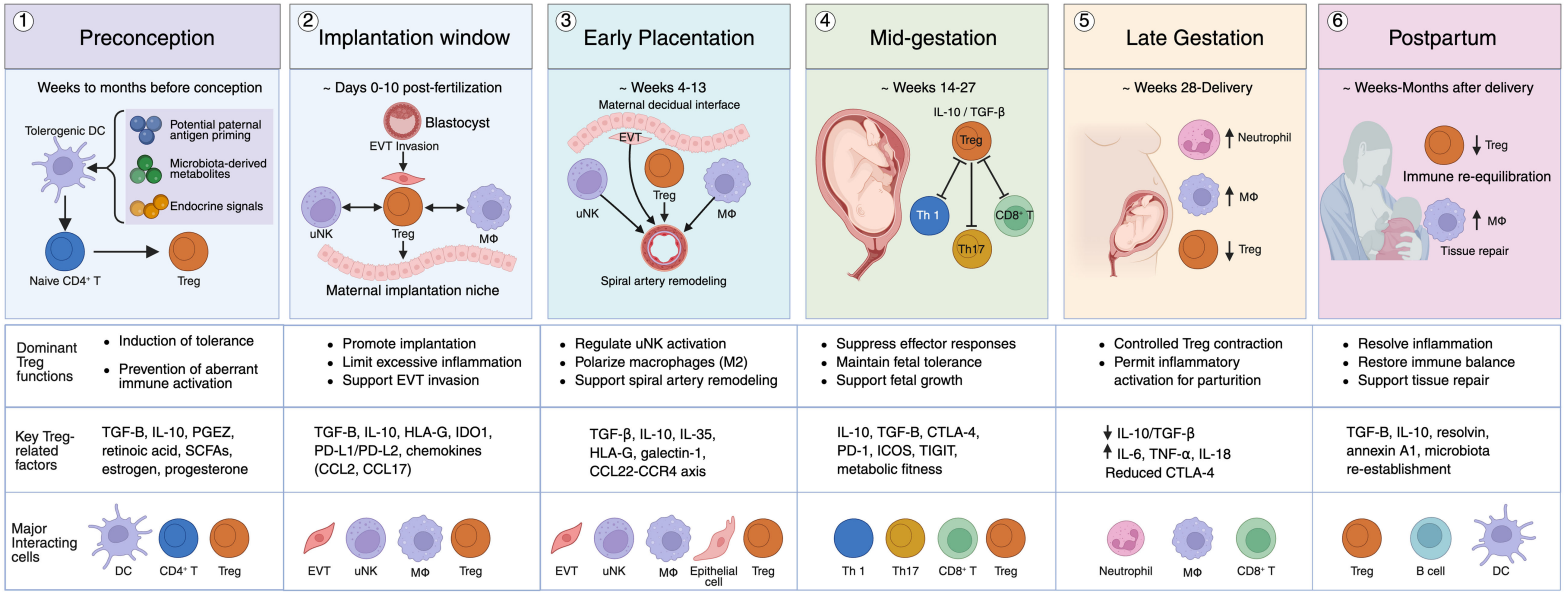
Stage-specific evolution of Treg biology and functions across pregnancy. This schematic summarizes the dynamic temporal evolution of regulatory T-cell (Treg) biology across pregnancy and the postpartum period, integrating stage-specific immunological functions, dominant regulatory mediators, and major interacting cellular partners (1). Preconception immune conditioning, including paternal antigen exposure, microbiota-derived signals, and endocrine influences that promote early Treg priming (2). Implantation-stage immune adaptation, where Tregs support establishment of a tolerogenic maternal implantation niche. (3) Early placentation, characterized by Treg-mediated regulation of uterine immune cells, trophoblast invasion, and spiral artery remodeling. (4) Mid-gestation maintenance of maternal-fetal tolerance through sustained suppression of effector immune responses. (5) Late gestational transition toward controlled inflammatory activation associated with parturition. (6) Postpartum immune re-equilibration, inflammatory resolution, and tissue repair. Representative stage-specific Treg-associated mediators and principal interacting cell populations are shown for each phase. Treg, regulatory T cell; EVT, extravillous trophoblast; uNK, uterine natural killer cell; DC, dendritic cell; MΦ, macrophage.

The stage-specific evolution of Treg biology across pregnancy underscores the dynamic nature of maternal immune adaptation, spanning preconception immune conditioning, implantation, placentation, maintenance of fetal tolerance, late gestational immune transition, and postpartum recovery. To integrate these temporal, mechanistic, and functional dimensions, **Figure 1** presents a stage-specific conceptual framework summarizing the evolving roles of Tregs, their major interacting cellular partners, and dominant immunoregulatory mechanisms across pregnancy and the postpartum period.

## 3 Beyond Treg abundance: what changes in disease?

Initial studies investigating Tregs in pregnancy disorders primarily focused on quantifying FOXP3^+^ T cells in peripheral blood or decidual tissues (22, 73). Although these studies provided important initial insights, it is now clear that changes in Treg abundance alone cannot fully explain immune dysregulation associated with pregnancy complications (74, 75). Instead, pregnancy disorders are characterized by multidimensional alterations in Treg biology, including changes in functional competence, lineage stability, and spatial distribution (58, 76).

Building on the stage-specific framework described above, Treg dysregulation in pregnancy disorders can be interpreted across four interrelated dimensions-quantitative abundance, functional competence, lineage stability, and spatial distribution-each contributing differently to disease pathogenesis. Dissecting these dimensions provides a more precise understanding of how Treg alterations drive immune imbalance and informs the development of mechanistically grounded biomarkers and therapeutic strategies (27, 77, 78).

### 3.1 Quantitative versus functional alterations

Reduced Treg frequency has been reported in multiple pregnancy complications, including recurrent pregnancy loss and preeclampsia; however, the magnitude and consistency of these findings vary across studies (6, 27, 79). Variability in study design, patient populations, gestational timing, and Treg identification strategies likely contributes to these discrepancies. Importantly, functional impairment of Tregs may occur independently of numerical changes, indicating that cell abundance alone is an incomplete measure of regulatory capacity (27, 80, 81).

Functionally, Treg dysfunction in pregnancy disorders is characterized by reduced suppressive capacity and disruption of regulatory signaling networks that normally constrain effector immune activation (12, 82). These alterations impair control of pro-inflammatory Th1 and Th17 responses, as well as cytotoxic CD8^+^ T-cell activity, thereby promoting immune activation at the maternal-fetal interface (19, 83). Representative functional alterations associated with Treg dysfunction include reduced FOXP3 expression, diminished production of immunoregulatory cytokines such as IL-10 and TGF-β, decreased expression of inhibitory receptors including CTLA-4, and increased effector-to-regulatory immune ratios (e.g., Th17/Treg), all of which have been linked to impaired immune tolerance in pregnancy disorders (84). Taken together, these findings indicate that assessment of Treg function, rather than enumeration alone, is essential for accurately interpreting immune dysregulation in pregnancy complications.

These observations further suggest that quantitative alterations represent only one dimension of Treg dysfunction, necessitating consideration of lineage stability and phenotypic plasticity as additional determinants of regulatory failure.

### 3.2 Lineage stability and Treg plasticity

Beyond functional impairment, disruption of Treg lineage stability represents a critical mechanism underlying immune dysregulation in pregnancy disorders (71, 85). Under physiological conditions, stable FOXP3 expression ensures sustained suppressive function; however, inflammatory environments may compromise this stability, leading to phenotypic plasticity (86, 87). Such instability can result in partial loss of regulatory activity or conversion into pro-inflammatory effector-like cells (84, 88).

Inflammatory stress conditions can compromise Treg lineage stability and promote phenotypic plasticity, particularly in settings characterized by chronic inflammation, tissue stress, or dysregulated inflammatory signaling (86, 89). Such conditions are frequently observed in pregnancy disorders associated with placental hypoxia, oxidative stress, or systemic inflammation (90–92). Under these pathological circumstances, Tregs may exhibit impaired suppressive function, reduced lineage stability, or altered phenotypic characteristics that contribute to dysregulated immune responses (13, 27, 93).

These findings position lineage instability as a key mechanistic link between environmental stress signals and loss of immune regulation. Understanding the factors that govern Treg stability during pregnancy may therefore provide critical insight into disease pathogenesis and identify opportunities for therapeutic intervention (13, 27, 93).

### 3.3 Tissue-resident versus circulating Tregs

In addition to quantitative and functional alterations, spatial distribution of Tregs represents a distinct and clinically relevant dimension of immune regulation. Tregs in the peripheral circulation differ substantially from those residing within the decidua and placental tissues, where local environmental cues shape their phenotype and function (14, 51). Compared with circulating peripheral Tregs, decidual Tregs constitute a functionally specialized population characterized by stronger suppressive capacity, increased expression of immunoregulatory molecules such as CTLA-4, PD-1, and ICOS, enhanced production of tolerogenic mediators including IL-10 and TGF-β, and tissue-adaptive retention programs that support persistence within the maternal-fetal microenvironment (9, 40).

In pregnancy disorders, alterations in decidual Treg populations may occur independently of changes in circulating Tregs (58). Impaired recruitment, defective local expansion, or altered retention of Tregs within the decidua may reduce their presence at the maternal-fetal interface, even when peripheral Treg numbers remain unchanged (50, 51). Conversely, systemic immune alterations may affect circulating Tregs without accurately reflecting local immune conditions within placental tissues (39, 94).

These observations underscore that spatial context is critical for interpreting Treg biology. Distinguishing between systemic and tissue-specific alterations is therefore essential for understanding disease mechanisms and for developing reliable biomarkers that accurately capture local immune dynamics (95, 96).

### 3.4 Molecular regulators of Treg function

The functional integrity of Tregs is governed by interconnected molecular pathways involving lineage-defining transcription factors, inhibitory receptors, cytokine signaling, and metabolic regulation (52, 97). FOXP3 remains central to Treg identity, while immune checkpoint molecules such as CTLA-4, TIGIT, and PD-1 mediate cell-cell interactions that suppress immune activation (12, 98). In parallel, cytokine-dependent mechanisms contribute to maintenance of immune tolerance through coordinated regulation of inflammatory responses (12, 52, 99).

In pregnancy disorders, dysregulation of these molecular pathways disrupts Treg function at multiple levels (17, 99). Reduced FOXP3 expression, altered checkpoint signaling, and impaired downstream regulatory networks can compromise suppressive capacity (100, 101). In addition, metabolic regulators including mTOR, AMP-activated protein kinase (AMPK), and hypoxia-inducible pathways have emerged as key determinants of Treg differentiation and stability (102, 103). Given the metabolically dynamic and often hypoxic nature of the placental environment, these pathways may represent critical nodes linking tissue stress to Treg dysfunction (104, 105).

Together, these molecular regulators integrate environmental signals with Treg function, providing a mechanistic basis for how diverse pathological stimuli converge to disrupt immune tolerance during pregnancy.

### 3.5 Technical challenges in defining Tregs

Interpretation of Treg alterations in pregnancy disorders is further complicated by methodological limitations in accurately identifying these cells (6, 13, 28, 74). Although FOXP3 expression is widely used as a defining marker, it can be transiently expressed by activated conventional T cells without conferring suppressive function (106, 107). Consequently, reliable identification of functional Tregs often requires additional markers, including CD25, CD127, and epigenetic signatures associated with stable FOXP3 expression (108, 109).

Variability in gating strategies, antibody panels, and tissue sampling approaches contributes to inconsistencies across studies (110). Moreover, differences between peripheral blood and decidual samples further complicate interpretation (111). Advances in high-dimensional immune profiling technologies, including single-cell transcriptomics and multi-parameter flow cytometry, are improving the resolution of Treg characterization (112). However, standardization of these approaches will be essential for translating Treg-based measurements into clinically meaningful biomarkers (113, 114).

A more fundamental unresolved challenge is whether bulk Treg measurements adequately capture the biologically relevant regulatory populations responsible for maternal-fetal tolerance. If antigen-responsive or fetal alloantigen-reactive Treg subsets represent the critical suppressive populations, aggregate quantification may obscure clinically meaningful deficiencies in rare but functionally important subpopulations (6, 67). Tissue localization further complicates interpretation, as circulating Treg profiles may not accurately reflect the abundance, positioning, or suppressive competence of decidual Tregs at the maternal-fetal interface (51, 111). Addressing this limitation will likely require greater analytical granularity, including single-cell profiling, spatial immune analysis, and approaches capable of resolving antigen-specific T-cell receptor repertoires (112).

From a conceptual perspective, these technical challenges highlight a broader issue: discrepancies in reported Treg alterations may reflect limitations in measurement rather than true biological differences. Addressing these limitations is therefore critical for accurately defining Treg dysfunction and advancing biomarker development.

## 4 Disease-specific versus shared Treg signatures across pregnancy disorders

A growing body of evidence indicates that Treg dysregulation is a common feature across multiple pregnancy complications (27, 115). However, these alterations are not uniform; instead, they reflect context-dependent perturbations across distinct dimensions of Treg biology, including abundance, functional competence, lineage stability, and spatial distribution (116, 117).

Rather than representing disease-specific phenomena in isolation, Treg alterations can be understood as variations along shared regulatory axes shaped by disease timing, inflammatory drivers, and tissue context. This perspective enables direct comparison across disorders and provides a framework for distinguishing common immunological mechanisms from condition-specific adaptations. Such comparative analysis is essential for identifying robust biomarkers and for guiding the development of mechanism-based therapeutic strategies (20, 28, 78, 118, 119).

### 4.1 Recurrent pregnancy loss and recurrent implantation failure

Recurrent pregnancy loss (RPL) and recurrent implantation failure (RIF) represent early-stage disorders characterized primarily by failure to establish immune tolerance at the maternal-fetal interface (120, 121). Within the proposed framework, these conditions are dominated by quantitative and functional deficits, including reduced Treg abundance and impaired suppressive activity, accompanied by expansion of pro-inflammatory T-cell responses (120–122).

A hallmark feature is the imbalance between Tregs and Th17 cells, reflecting insufficient regulatory control over inflammatory signaling during implantation (19, 120, 123). In addition, impaired recruitment or local expansion of Tregs within the decidua highlights a spatial dimension of dysfunction, where insufficient Treg presence at the maternal-fetal interface compromises tolerance establishment (6, 9, 14).

Pro-inflammatory cytokine environments enriched in IL-6, IL-17, and TNF-α further destabilize Treg function, linking inflammatory signaling to both functional impairment and potential lineage instability (63, 81, 124). Although these mechanisms are not unique to RPL or RIF, their occurrence during the implantation window defines these disorders as failures of early immune programming rather than breakdown of established tolerance.

### 4.2 Preeclampsia and hypertensive disorders of pregnancy

Preeclampsia represents a later-stage disorder in which Treg dysregulation occurs within the context of abnormal placental development and systemic inflammation (125, 126). In contrast to early implantation disorders, preeclampsia is characterized by combined perturbations across functional, spatial, and vascular-coupled dimensions of Treg biology.

Reduced Treg abundance in both peripheral and decidual compartments has been reported (14, 127); however, functional impairment appears to play a more prominent role, with insufficient suppression of Th1- and Th17-mediated inflammatory responses (128, 129). These alterations contribute to defective spiral artery remodeling and impaired placental perfusion, linking immune dysregulation to vascular pathology (130).

Importantly, preeclampsia also illustrates disruption of immune-vascular coupling, in which Treg dysfunction simultaneously affects local placental processes and systemic maternal inflammation. Altered recruitment and positioning of Tregs within the decidua further emphasize a spatial component, while systemic immune activation extends the impact beyond the maternal-fetal interface (20, 124, 131).

### 4.3 Spontaneous PTL

Spontaneous PTL is often associated with inflammatory processes that activate uterine contractions and premature cervical remodeling (132, 133). Within the Treg framework, PTL is characterized predominantly by functional impairment and inflammatory override, with relatively preserved Treg numbers but reduced suppressive capacity (19, 27, 53). Elevated levels of pro-inflammatory cytokines, including IL-1β, IL-6, and IL-17, create an environment that destabilizes regulatory control and amplify inflammatory signaling pathways (134, 135). This inflammatory amplification promotes uterine activation and premature cervical remodeling, ultimately triggering early delivery (63, 136). Unlike implantation-related disorders, PTL reflects breakdown of an already established regulatory state, highlighting the importance of sustained Treg function in maintaining immune equilibrium during later stages of pregnancy.

### 4.4 Placental inflammatory lesions and villitis

Placental inflammatory lesions, including villitis of unknown etiology and chronic deciduitis, represent localized breakdown of immune tolerance within placental tissues (137, 138). These conditions are primarily characterized by spatially restricted Treg dysfunction, where insufficient regulatory control permits immune cell infiltration into placental structures. Reduced Treg function or inadequate decidual Treg presence may permit pathogenic effector T cell activation and localized inflammatory injury within villous tissues (9). This localized immune dysregulation is associated with adverse outcomes, including fetal growth restriction and preterm birth (139, 140). These lesions illustrate how disruption of Treg-mediated control within specific tissue niches can drive pathology independently of systemic immune changes, reinforcing the importance of spatial context in Treg biology.

### 4.5 Polycystic ovary syndrome-associated endometrial dysfunction and pregnancy complications

Polycystic ovary syndrome (PCOS) represents a distinct context in which Treg dysregulation arises within a background of metabolic and endocrine disturbance (141, 142). In this setting, alterations in Treg biology are closely linked to lineage stability and environmental modulation, rather than purely quantitative deficits. Chronic low-grade inflammation, insulin resistance, and altered cytokine signaling contribute to disruption of Treg differentiation and stability, leading to impaired immune regulation within the endometrium (143, 144). These changes affect endometrial receptivity and early pregnancy tolerance, indicating that metabolic stress can influence Treg function through both functional and stability-related mechanisms. PCOS therefore highlights an additional dimension of Treg dysregulation, in which systemic metabolic conditions reshape immune regulation prior to and during early pregnancy.

### 4.6 Shared and distinct immunological patterns

Across pregnancy disorders, Treg dysregulation converges on a limited set of shared immunological features, including reduced suppressive capacity, enhanced pro-inflammatory signaling, and imbalance between regulatory and effector T-cell populations (17, 41). However, the dominant dimension of dysfunction differs depending on disease context and timing.

Early-stage disorders such as RPL and RIF are primarily driven by deficits in Treg abundance and function that impair tolerance establishment (121, 122). In contrast, later-stage conditions such as preeclampsia and PTL reflect breakdown of established regulatory networks, involving functional impairment, spatial redistribution, and inflammatory amplification (145, 146). Meanwhile, conditions such as PCOS introduce additional layers of metabolic and stability-related disruption that influence Treg biology prior to implantation (37, 147).

A key insight from this comparative framework is that Treg dysregulation is not disease-specific but context-dependent, with different combinations of quantitative, functional, stability, and spatial alterations underlying distinct clinical phenotypes (148). This has important implications for both biomarker development and therapeutic design. Static measurements of Treg frequency are unlikely to capture the complexity of immune dysregulation; instead, multi-dimensional and context-aware approaches will be required (30).

**Figure 2:**
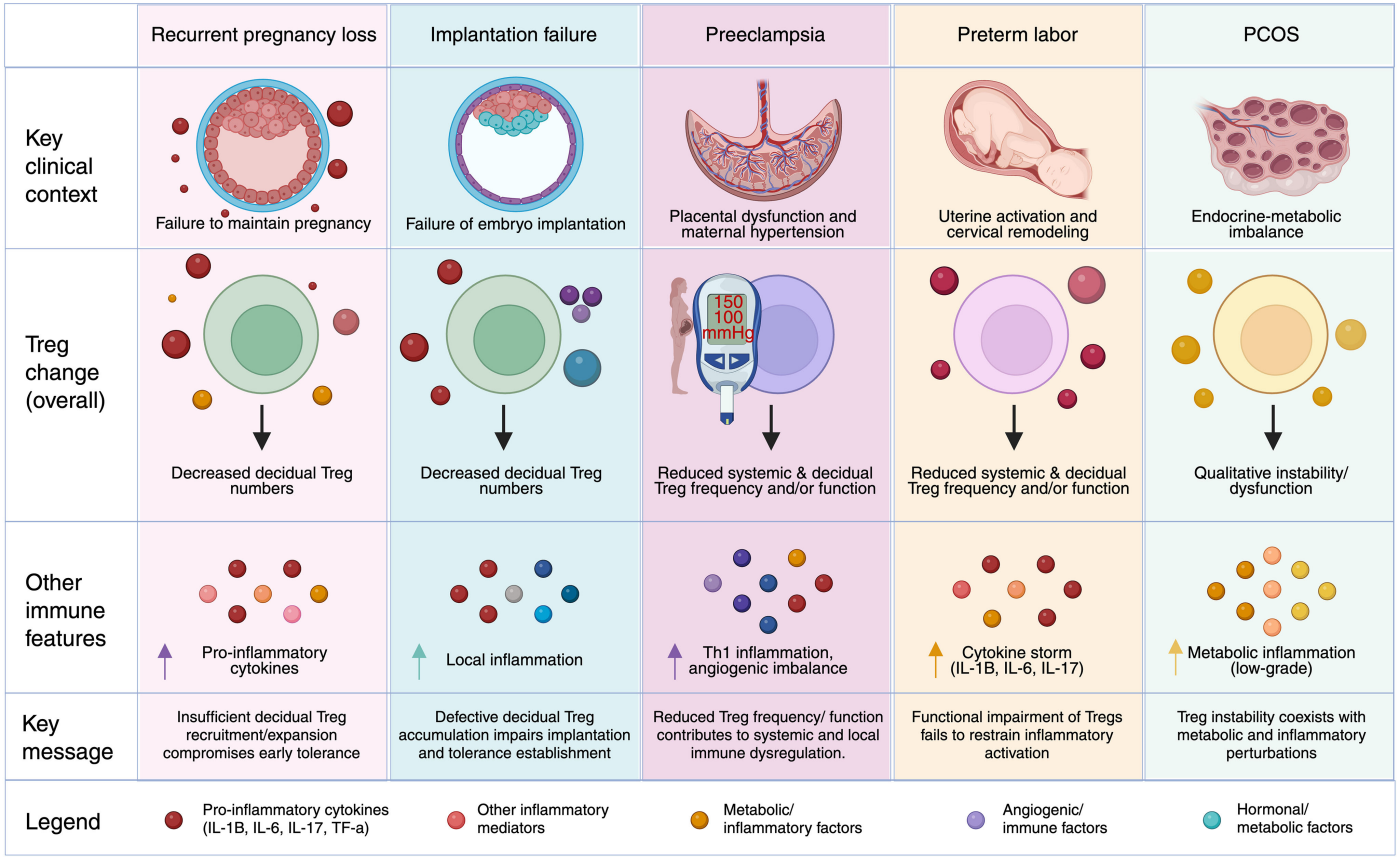
Comparative patterns of Treg dysregulation across pregnancy disorders. This schematic presents a comparative overview of Treg dysregulation across major pregnancy complications, including RPL, implantation failure, preeclampsia, PTL, and PCOS-associated reproductive dysfunction. For each condition, the figure summarizes four comparative dimensions: clinical context, overall Treg alterations, associated immune features, and key mechanistic interpretation. Early pregnancy disorders such as RPL and implantation failure are characterized predominantly by impaired establishment of maternal immune tolerance associated with reduced decidual Treg accumulation. Preeclampsia reflects combined systemic and local immune dysfunction with impaired Treg abundance and/or function linked to inflammatory and vascular imbalance. PTL is characterized primarily by functional Treg impairment within a heightened pro-inflammatory environment, whereas PCOS-associated reproductive dysfunction is associated with Treg instability in the setting of chronic metabolic inflammation. Colored circles represent representative inflammatory, angiogenic, metabolic, or hormonal mediators rather than specific cell populations. Arrows indicate relative directional changes in Treg abundance or associated immune activity.

These considerations suggest that effective immunomodulatory strategies must be stage-specific and mechanism-informed, targeting tolerance induction in early pregnancy, stabilization of regulatory networks during gestation, or suppression of excessive inflammation in later stages. To facilitate visualization of these relationships, **Figure 2** provides a schematic representation of shared and disorder-specific patterns of Treg dysregulation across pregnancy complications.

**Table 1** summarizes representative studies across pregnancy disorders, highlighting how different dimensions of Treg dysregulation-particularly functional impairment, spatial alterations, and lineage instability are variably captured depending on study design and measurement approaches.

**Table 1:** Key studies evaluating Treg alterations in pregnancy disorders.

Disease	Sample type	Treg marker	Key findings	References
Recurrent pregnancy loss (RPL)	Peripheral blood, decidua	CD4^+^CD25^high^ FOXP3^+^	Reduced decidual Tregs and FOXP3 expression, indicating impaired immune tolerance	(51, 121)
Early pregnancy failure/miscarriage	Decidua or peripheral blood	FOXP3-related Treg signatures	Reduced FOXP3-associated Treg function, indicating immune-mediated pregnancy loss	(17, 41)
Preeclampsia	Peripheral blood, decidua, Placenta	Helios^+^/Helios^-^ FOXP3^+^ Tregs; FOXP3, CCL22	Impaired expansion of adaptive Tregs, Th1-skewed inflammation, and reduced Treg recruitment associated with altered chemokine signaling	(149, 150)
Term vs PTL	Peripheral blood	CD4^+^ CD127^low^CD25^+^FOXP3^+^	Preserved Treg numbers but reduced suppressive function	(22, 108)
Spontaneous PTL	Peripheral blood	ICOS^+^/ICOS^-^ Tregs	Impaired differentiation of recent thymic emigrant Tregs	(19, 27)
Idiopathic preterm birth	Maternal-fetal interface	Functional Tregs, Tc17	Reduced Treg function with increased Tc17 responses	(151, 152)
Chronic histiocytic intervillositis (CHI/CIUE)	Decidua, intervillous space	FOXP3^+^ Tregs	Increased Treg infiltration, suggesting lesion-specific or compensatory response	(153, 154)
PCOS-associated pregnancy dysfunction	Endometrium	Treg/Th1/Th17 subsets	T-cell imbalance with altered Treg-associated immune regulation	(155, 156)

Human Tregs are commonly defined as CD4^+^CD25^high^CD127^low/−^FOXP3^+^ T cells, a phenotype that improves specificity for suppressive Tregs in flow cytometry analyses.

## 5 Tregs as orchestrators of the maternal-fetal immune network

Immune tolerance during pregnancy is not governed by a single immune cell population but rather by a coordinated network of cellular interactions within the maternal-fetal interface (53, 157). Within this system, Tregs function as central integrators that couple immune regulation with tissue remodeling and vascular adaptation (158). Through direct cell-cell interactions and context-dependent signaling, Tregs influence the activity of multiple immune and non-immune cell populations, including uNK cells, macrophages, trophoblasts, and decidual stromal cells (14, 53). These coordinated interactions establish a permissive yet tightly regulated microenvironment that supports fetal tolerance while enabling placental development and structural adaptation (40, 159).

### 5.1 Treg-uNK cell interactions

uNK cells represent the dominant immune population within the decidua during early pregnancy and are specialized for tissue remodeling rather than cytotoxic defense (59, 160). Their functional phenotype must be tightly controlled to prevent trophoblast damage while supporting vascular development (8, 161). Tregs regulate uNK cell activity through integrated cytokine-dependent and contact-mediated mechanisms that constrain activation and promote a pro-angiogenic phenotype (12, 52). In addition, suppression of upstream inflammatory T-cell responses indirectly limits NK-cell cytotoxicity by reducing pro-inflammatory signaling inputs (14, 162). Through these mechanisms, Tregs maintain the balance between immune restraint and vascular support that is essential for placental development.

### 5.2 Treg-macrophage crosstalk

Decidual macrophages play central roles in tissue remodeling, debris clearance, and immune regulation, often adopting an anti-inflammatory M2-like phenotype that supports pregnancy maintenance (58, 163, 164). Tregs actively shape this phenotype through cytokine-mediated signaling, particularly IL-10-dependent pathways, which promote the differentiation and stabilization of tolerogenic macrophage populations (4, 165). These macrophages, in turn, reinforce immune tolerance by producing anti-inflammatory mediators and growth factors that support placental development and suppress excessive immune activation (10, 166). Disruption of this bidirectional interaction shifts macrophage polarization toward pro-inflammatory states, linking Treg dysfunction to inflammatory microenvironments that compromise placental integrity (167, 168).

### 5.3 Treg-trophoblast communication

Trophoblast cells mediate the interface between maternal tissues and the developing fetus and are central to placental formation and vascular remodeling (169, 170). Their interaction with immune cells must be precisely regulated to allow controlled invasion without triggering immune rejection (171). Tregs contribute to this balance by suppressing local inflammatory responses and creating conditions permissive for trophoblast invasion (14, 51, 58). Proposed trophoblast-Treg communication likely involves both soluble and contact-dependent regulatory mechanisms. Trophoblast-derived mediators such as HLA-G, TGF-β, IL-10, and galectin-1 may promote Treg differentiation, recruitment, and functional maintenance, while chemokine-mediated signaling may contribute to localized enrichment of regulatory populations at the maternal-fetal interface. In addition, direct cell-cell immune regulatory interactions, including checkpoint-associated signaling pathways, may further reinforce localized immune tolerance (39, 172). This bidirectional communication links immune regulation directly to placental development.

### 5.4 Treg-decidual stromal cell interactions

Decidual stromal cells provide structural and immunological support at the maternal-fetal interface by producing cytokines, chemokines, and growth factors that regulate immune cell recruitment and differentiation (173, 174). Decidual stromal cells can also promote Treg expansion and stabilization through the secretion of immunomodulatory factors, including TGF-β and prostaglandins (173, 174). In turn, Tregs actively contribute to maintenance of the tolerogenic stromal microenvironment through immunoregulatory mediators such as IL-10 and TGF-β, which may suppress local inflammatory signaling and reinforce stromal immune-regulatory functions. By limiting effector immune activation and sustaining anti-inflammatory conditions, this reciprocal interaction stabilizes the local immune niche while supporting ongoing tissue remodeling and maternal-fetal tolerance (17, 53).

### 5.5 Implications for spiral artery remodeling and placental development

Spiral artery remodeling is a critical process that ensures adequate blood supply to the developing placenta and requires coordinated interactions among trophoblasts, uNK cells, macrophages, and stromal cells (39, 175). Tregs indirectly regulate this process by modulating the activity and interactions of these cell populations (14, 58). By maintaining immune equilibrium and preventing excessive inflammation, Tregs enable trophoblast invasion and vascular remodeling to proceed without immune-mediated disruption (17, 159). Conversely, impaired Treg function can lead to dysregulated immune responses that compromise placental development and vascular adaptation, contributing to conditions such as preeclampsia and fetal growth restriction (14, 20, 176). These network-level interactions reinforce the concept that Tregs function as central coordinators of immune-tissue crosstalk rather than isolated suppressive cells. Disruption of these interactions may therefore have system-level consequences, simultaneously affecting immune regulation, vascular remodeling, and placental function. This perspective further supports the need to interpret Treg alterations within an integrated, multi-cellular framework.

## 6 Upstream drivers of Treg dysfunction

Treg abundance, function, stability, and spatial distribution during pregnancy are shaped by a network of systemic and local regulatory inputs (41, 173). Rather than acting independently, hormonal, metabolic, microbial, and inflammatory signals converge to modulate Treg differentiation, lineage stability, and suppressive capacity (53, 177). Disruption of these upstream regulators can therefore shift Treg biology across multiple dimensions simultaneously, amplifying immune dysregulation at the maternal-fetal interface and contributing to pregnancy complications (41).

From a conceptual perspective, these upstream drivers do not simply reduce Treg numbers but reshape the regulatory landscape by altering functional competence, destabilizing lineage identity, and perturbing tissue-specific immune organization. Understanding how these factors integrate to influence Treg biology is essential for identifying mechanistically grounded biomarkers and for developing targeted therapeutic strategies (20).

### 6.1 Hormonal regulation

Pregnancy is characterized by profound endocrine changes that shape maternal immune adaptation. Endocrine signaling represents a major regulator of Treg biology during pregnancy, linking systemic physiological changes to immune tolerance at the maternal-fetal interface. Hormones such as progesterone, estrogen, and human chorionic gonadotropin (hCG) have been shown to promote immune tolerance by supporting Treg expansion, functional competence, and lineage stability (49, 173). Progesterone exerts particularly strong immunomodulatory effects by inducing tolerogenic dendritic cells and promoting Treg differentiation, in part through progesterone-induced blocking factor (PIBF)-mediated signaling pathways (178, 179). Estrogen further supports Treg expansion and enhances FOXP3 expression in a dose-dependent manner, contributing to sustained regulatory activity (180). Disruption of hormonal signaling as observed in endocrine disorders such as PCOS can impair Treg stability and shift immune balance toward pro-inflammatory states, linking systemic endocrine dysfunction to defective immune tolerance and impaired implantation or placental development (181).

### 6.2 Microbiome and microbial metabolites

The maternal microbiome represents a key regulator of systemic and local immune homeostasis through the production of immunomodulatory metabolites (182). Short-chain fatty acids (SCFAs), including acetate, propionate, and butyrate, promote Treg differentiation by enhancing FOXP3 expression and stabilizing epigenetic programs associated with lineage commitment (66, 183–185). Dysbiosis disrupts this regulatory axis, altering metabolite availability and promoting inflammatory signaling that can impair Treg differentiation and suppressive function (186). Such alterations have been linked to pregnancy complications including preeclampsia, gestational diabetes, and recurrent pregnancy loss (187). Conceptually, the microbiome-Treg axis illustrates how environmental signals can regulate immune tolerance through metabolic-epigenetic coupling, influencing both systemic immune tone and local immune conditions within the placenta (121, 188).

### 6.3 Hypoxia and immunometabolism

Metabolic conditions within the placental microenvironment represent a critical determinant of Treg function and stability. Physiological hypoxia during early pregnancy supports trophoblast invasion and vascular remodeling; however, pathological hypoxia resulting from impaired placental perfusion introduces metabolic stress that disrupts immune regulation (186, 189, 190). Hypoxia regulates Treg biology through hypoxia-inducible factors (HIFs) and metabolic signaling pathways, including mTOR and AMPK, which govern cellular energy utilization and T-cell differentiation (117, 191). Under conditions of oxidative stress or nutrient imbalance, these pathways can impair Treg suppressive function and promote phenotypic instability (191, 192). These findings highlight a direct link between tissue metabolic state and immune regulation, positioning immunometabolism as a key interface through which placental dysfunction can drive Treg dysregulation in disorders such as preeclampsia and fetal growth restriction (193).

### 6.4 Inflammatory cytokine circuits

Pro-inflammatory cytokines represent central mediators that directly modulate Treg function and lineage stability (76). Cytokines such as IL-6, IL-1β, and TNF-α disrupt Treg differentiation and can destabilize FOXP3 expression, promoting a shift toward pro-inflammatory T-cell phenotypes, including Th17 cells (57, 86, 95, 194, 195). These cytokine-driven circuits operate as amplification loops, in which inflammatory signaling both impairs regulatory mechanisms and enhances effector responses. For example, IL-6 signaling inhibits Treg differentiation while simultaneously promoting Th17 expansion, reinforcing immune imbalance (52, 196). Such feedback mechanisms are particularly relevant in pregnancy disorders characterized by chronic inflammation, where sustained cytokine signaling can progressively erode Treg-mediated immune control and exacerbate placental dysfunction (197, 198).

### 6.5 Genetic and epigenetic influences

Genetic and epigenetic mechanisms provide an additional layer of regulation governing Treg differentiation and stability. Variants in genes associated with immune regulation, including FOXP3, have been linked to susceptibility to pregnancy complications such as recurrent pregnancy loss (199, 200). Epigenetic regulation including DNA methylation at the FOXP3 locus, histone modifications, and microRNA-mediated control plays a critical role in maintaining Treg lineage commitment and functional integrity (201, 202). These mechanisms are highly sensitive to environmental inputs, including inflammation, metabolic stress, and microbial-derived signals (76, 203). Thus, genetic predisposition and environmentally induced epigenetic changes converge to shape Treg stability, providing a mechanistic basis for inter-individual variability in immune tolerance during pregnancy.

### 6.6 Maternal systemic disease states

Maternal systemic conditions, including metabolic disorders, autoimmune diseases, and chronic inflammatory states, further modulate Treg biology by altering the systemic immune environment (115, 204). Obesity and insulin resistance are associated with persistent low-grade inflammation that impairs Treg differentiation and function (205, 206). Autoimmune diseases may introduce additional complexity, although their effects during pregnancy are heterogeneous and disease specific. In some contexts, pre-existing immune dysregulation may involve impaired Treg suppressive capacity, altered effector-regulatory immune balance, dysregulated inflammatory cytokine signaling, or defective recruitment and maintenance of regulatory populations at the maternal-fetal interface, thereby potentially compromising appropriate maternal adaptation to fetal alloantigens (207, 208).

These systemic factors interact with local immune processes at the maternal-fetal interface, amplifying dysregulation across multiple dimensions of Treg biology. From a translational perspective, this interaction highlights the importance of considering maternal health status as a determinant of immune regulation during pregnancy and supports the development of personalized approaches to risk assessment and therapeutic intervention (28, 74).

Collectively, these upstream drivers illustrate that Treg dysregulation arises from the convergence of endocrine, metabolic, microbial, inflammatory, and genetic inputs rather than from isolated perturbations. These factors interact across systemic and local scales to reshape Treg function, stability, and spatial organization, ultimately determining the balance between immune tolerance and inflammation. This integrated perspective reinforces the need to interpret Treg alterations within a multi-dimensional regulatory framework and highlights potential intervention points for restoring immune homeostasis during pregnancy.

## 7 Emerging technologies redefining Treg biology in pregnancy

Advances in high-dimensional immune profiling are fundamentally reshaping our understanding of Treg biology at the maternal-fetal interface (209). Traditional approaches including bulk transcriptomics and conventional flow cytometry have provided foundational insights but are limited in their ability to resolve cellular heterogeneity, spatial organization, and functional diversity (9, 210). Emerging technologies now enable interrogation of Tregs across multiple dimensions, including transcriptional identity, spatial localization, lineage stability, and interaction networks (33, 211).

From a conceptual perspective, these approaches are shifting the field from static enumeration of Tregs toward a multi-dimensional framework that integrates function, stability, and spatial context. This transition is particularly important for pregnancy, where immune regulation is highly dynamic and tissue specific. By resolving Treg heterogeneity and microenvironmental interactions, these technologies provide a foundation for redefining immune biomarkers and uncovering mechanisms of disease (12, 212).

### 7.1 Single-cell transcriptomics

Single-cell RNA sequencing (scRNA-seq) enables high-resolution analysis of cellular heterogeneity within placental and decidual tissues (213, 214). Application of this technology has revealed that Tregs are not a uniform population but consist of multiple subpopulations with distinct transcriptional programs, activation states, and functional capacities (211, 215). Decidual Tregs exhibit specialized gene expression profiles characterized by enhanced expression of immunoregulatory molecules, including FOXP3, CTLA-4, and IL-10, as well as chemokine receptors that support tissue residency and local immune suppression (9, 216). In addition, single-cell analyses have demonstrated dynamic changes in Treg composition across gestation, reflecting evolving immune requirements during implantation, placental development, and pregnancy maintenance (17, 210). Importantly, single-cell approaches also enable reconstruction of gene regulatory networks governing Treg differentiation and stability, providing mechanistic insight into how upstream drivers such as inflammation and metabolic stress reshape Treg function in pregnancy disorders (28, 58, 212).

### 7.2 Spatial transcriptomics and spatial proteomics

While single-cell sequencing defines cellular identity, it lacks spatial context due to tissue dissociation (217). Spatial transcriptomics and spatial proteomics overcome this limitation by preserving tissue architecture and enabling mapping of gene and protein expression within intact microenvironments (218). These technologies have revealed that immune regulation at the maternal-fetal interface is spatially organized, with Tregs localized to specific niches associated with trophoblast invasion and vascular remodeling (14, 33, 40, 219, 220). Such spatial positioning suggests that Treg function is tightly coupled to local tissue processes, including angiogenesis and immune tolerance. Spatial profiling also provides a powerful approach to identify localized immune dysregulation in pregnancy complications. Alterations in Treg distribution, density, or activation state within specific placental regions may serve as early indicators of pathological processes (17, 221). These findings reinforce the concept that spatial context is a critical determinant of Treg function.

### 7.3 Multi-omics integration

Integration of multi-layered biological data including genomics, epigenomics, transcriptomics, proteomics, and metabolomics enables a systems-level understanding of immune regulation during pregnancy (222, 223). These approaches are particularly relevant for Tregs, whose identity and function are tightly regulated by epigenetic and metabolic mechanisms (224, 225). Epigenetic regulation at the FOXP3 locus, including DNA methylation and histone modifications, plays a central role in maintaining Treg lineage stability (226). Integration of epigenomic and transcriptomic data allows identification of regulatory circuits controlling Treg differentiation and functional resilience within the maternal-fetal interface (220, 227). Metabolic profiling further links environmental conditions such as oxygen availability, nutrient status, and microbial metabolites to Treg function through immunometabolic pathways (228, 229). Together, these multi-omics approaches provide a comprehensive framework for understanding how genetic, environmental, and metabolic factors converge to regulate Treg biology in both healthy and pathological pregnancies (230).

### 7.4 Functional ex vivo immune assays

While omics-based technologies provide detailed molecular and phenotypic characterization, functional assays remain essential for directly assessing Treg suppressive capacity and stability (212). Ex vivo assays using peripheral or decidual immune cells enable quantification of Treg-mediated suppression of effector T-cell proliferation and cytokine production (231). Advances in high-dimensional flow cytometry and mass cytometry (CyTOF) now allow simultaneous assessment of multiple functional and signaling markers, providing deeper insight into Treg activation states and regulatory pathways (232). Integration of functional assays with omics data is therefore critical for linking molecular signatures to biological activity and for validating candidate biomarkers.

### 7.5 Limitations and future directions

Despite rapid technological progress, several challenges remain in translating high-dimensional immune profiling into clinically actionable biomarkers. Variability in sample collection, tissue processing, and data analysis can limit reproducibility across studies, while relatively small cohort sizes constrain generalizability (233). Future progress will depend on the integration of multi-omics and spatial technologies with longitudinal clinical data, enabling identification of predictive immune signatures that precede clinical manifestations of pregnancy complications (33, 234). Standardization of experimental and analytical approaches will be essential for enabling cross-study comparisons and for advancing biomarker validation. Collectively, these emerging technologies are redefining Treg biology from a static cell population to a dynamic, spatially organized, and functionally heterogeneous system. By integrating single-cell, spatial, and multi-omics approaches with functional validation, it is now possible to capture the full complexity of Treg regulation across gestation. This technological shift provides a foundation for developing multi-dimensional biomarkers that reflect not only Treg abundance, but also functional competence, stability, and spatial context key features required for translating Treg biology into clinical applications.

## 8 Are Tregs clinically useful biomarkers?

Given the central role of Tregs in maintaining maternal-fetal immune tolerance, considerable interest has focused on their potential as clinical biomarkers for pregnancy disorders (9). An effective biomarker should enable early detection of pathological processes, predict disease risk, and guide therapeutic decision-making (235). While numerous studies have reported associations between Treg alterations and pregnancy complications, translation into clinical practice remains limited (236). A central challenge lies in determining which dimensions of Treg biology quantitative abundance, functional competence, lineage stability, or spatial distribution are most clinically informative, and how these features can be integrated into reproducible and scalable biomarker frameworks. Importantly, no single parameter fully captures Treg-mediated immune regulation, suggesting that multi-dimensional approaches are required.

### 8.1 Circulating versus decidual Tregs

A fundamental limitation in Treg-based biomarker development is the disconnect between systemic immune measurements and local immune activity at the maternal-fetal interface (27, 237). Circulating Tregs are readily accessible and therefore attractive for clinical monitoring and reduced peripheral Treg frequencies have been reported in pregnancy complications such as preeclampsia and recurrent pregnancy loss (27, 238). However, peripheral measurements do not consistently reflect immune dynamics within placental tissues (239). Decidual Tregs exhibit specialized phenotypes and play distinct roles in regulating local immune responses (9, 215). In some cases, alterations in decidual Treg populations occur independently of systemic changes (14, 58).

This divergence highlights a key conceptual issue: circulating Tregs primarily reflect systemic immune tone, whereas decidual Tregs capture local regulatory function. Effective biomarker strategies must therefore account for both compartments rather than relying on either alone. Quantitatively, circulating Tregs are commonly assessed as frequencies within peripheral CD4^+^ T-cell compartments, whereas decidual Tregs often exhibit local enrichment, distinct activation phenotypes, and enhanced suppressive function at the maternal-fetal interface. In pathological pregnancies, discordance between peripheral and tissue-associated Treg alterations further underscores the importance of compartment-specific interpretation (9, 51, 111).

### 8.2 Immune ratios and functional signatures

Beyond absolute Treg abundance, composite immune metrics and functional markers provide a more informative assessment of immune balance. Ratios such as Th17/Treg and Th1/Treg reflect the dynamic equilibrium between regulatory and effector responses and have been associated with inflammatory states in pregnancy disorders (19, 22, 51, 81, 240). Functional markers including FOXP3 expression, inhibitory receptor signaling (CTLA-4, PD-1), and cytokine production offer insight into Treg suppressive capacity beyond simple cell counts (12, 99). Advances in transcriptomic and proteomic profiling are further enabling identification of gene expression signatures that define functional Treg states (241).

**Figure 3:**
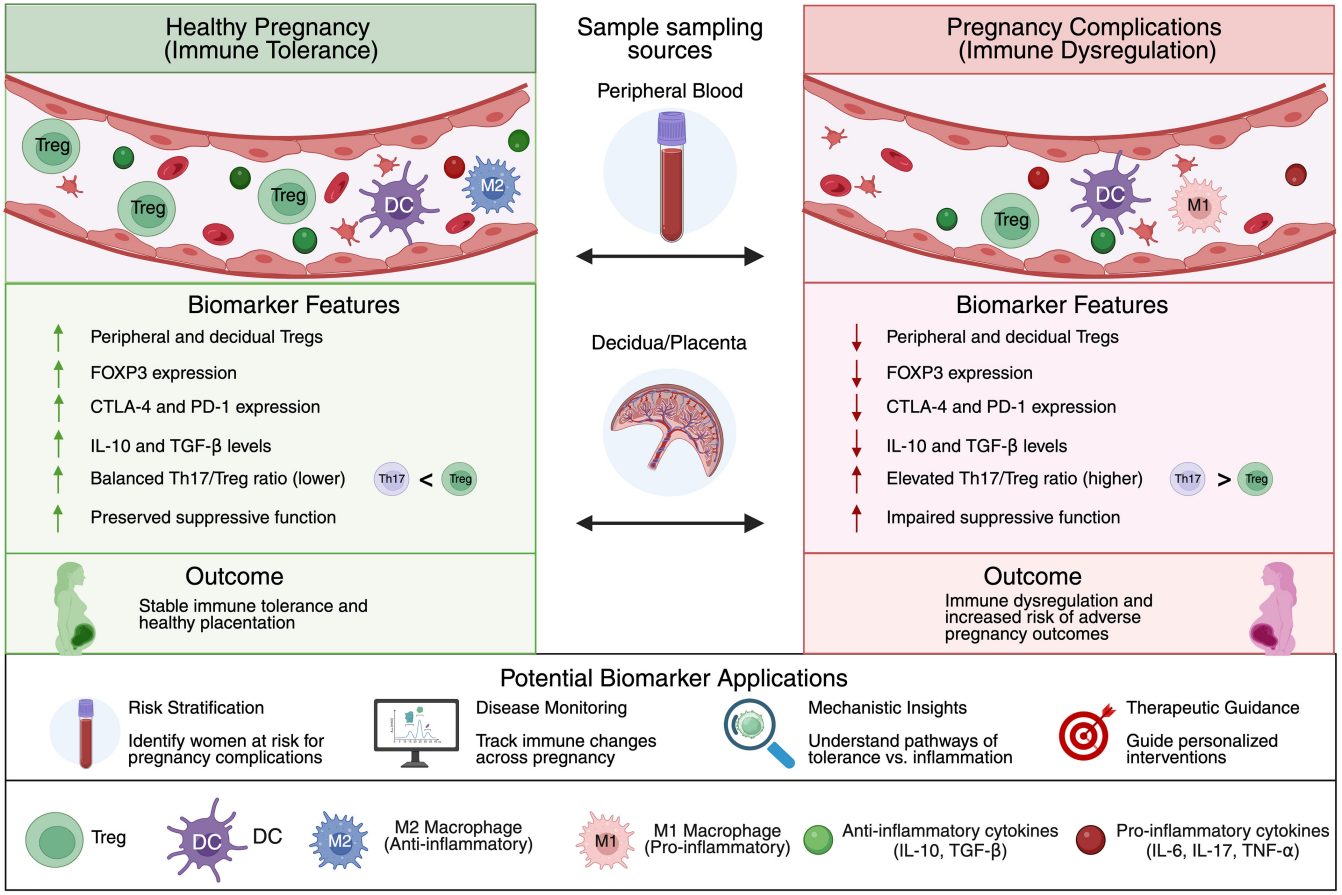
Treg-associated biomarker landscapes in healthy pregnancy versus pregnancy complications. This schematic illustrates the distinction and complementarity between circulating and tissue-resident Tregs as biomarkers in pregnancy disorders. Circulating Tregs in peripheral blood are readily accessible and may serve as predictive biomarkers, with alterations such as reduced Treg frequency and increased Th17/Treg ratios reflecting systemic immune imbalance that can precede clinical manifestations. However, peripheral measurements do not fully capture immune dynamics at the maternal-fetal interface. In contrast, decidual Tregs within placental tissues exhibit functionally specialized phenotypes and play central roles in local immune regulation, providing mechanistic insight into disease processes despite limited clinical accessibility. Key molecular and functional markers, including FOXP3, CTLA-4, PD-1, IL-10, and immune ratios such as Th17/Treg, are highlighted as indicators of Treg activity. Collectively, this framework underscores the need to integrate systemic and tissue-specific immune profiling to improve biomarker accuracy, risk stratification, and mechanistic interpretation of pregnancy complications.

Together, these approaches emphasize that functional competence, rather than numerical abundance alone, is a critical determinant of biomarker performance. This conceptual distinction is summarized in **Figure 3**.

### 8.3 From biomarker discovery to clinical implementation

Despite the identification of numerous candidate Treg-related biomarkers, clinical translation remains constrained by gaps in validation, standardization, and implementation (242). Biomarker development requires a structured pipeline encompassing discovery, validation, and clinical deployment, yet most studies remain limited to early-stage discovery without sufficient replication in independent cohorts (118, 243–245).

A key challenge lies in balancing biological resolution with clinical feasibility. Simple quantitative markers such as circulating Treg frequency and immune ratios are readily accessible but lack specificity. In contrast, functional biomarkers, including cytokine production and checkpoint receptor expression, provide greater mechanistic insight but require more complex assays (12, 246). High-dimensional signatures derived from single-cell or spatial profiling offer the greatest biological depth but remain difficult to implement in routine clinical settings due to cost, technical complexity, and lack of standardization (33, 247).

Methodological variability further complicates translation. Differences in marker selection, flow cytometry gating strategies, and sample processing protocols contribute to inconsistent findings across studies, while discrepancies between peripheral and tissue-derived measurements limit interpretability (248). As a result, the primary bottleneck in biomarker development is not the identification of candidate markers but the lack of reproducibility, scalability, and harmonization required for clinical adoption.

Addressing these challenges will require standardized protocols, integration of multi-dimensional immune features, and validation across large, well-characterized cohorts. Such efforts are essential for translating Treg-based immune signatures into clinically actionable tools.

### 8.4 Diagnostic versus prognostic applications

The clinical utility of Treg-related biomarkers depends on their intended application. Diagnostic biomarkers identify established disease, whereas prognostic biomarkers aim to predict disease risk prior to clinical manifestation (249, 250). In pregnancy disorders, prognostic biomarkers are particularly valuable, as many complications originate during early placental development but are diagnosed later in gestation (20, 207, 251). From a mechanistic perspective, these two applications may rely on different dimensions of Treg biology. Diagnostic biomarkers often reflect overt immune dysregulation, including reduced suppressive capacity and heightened inflammatory signaling. In contrast, prognostic biomarkers are more likely to capture early deviations in Treg abundance, stability, or immune balance before clinical symptoms emerge (252, 253).

This distinction underscores the importance of temporal context in biomarker interpretation, as immune alterations that are predictive at early gestational stages may differ from those observed in established disease. However, robust validation across diverse populations and longitudinal cohorts is required before these biomarkers can be reliably integrated into clinical risk stratification models.

### 8.5 Gestational timing and longitudinal dynamics

A critical consideration in Treg-based biomarker development is the dynamic nature of immune regulation across gestation (6, 27). Treg populations undergo stage-specific changes that reflect evolving immunological requirements (39, 254). Consequently, biomarker interpretation must account for gestational timing. Single time-point measurements may be insufficient to distinguish physiological immune adaptation from early pathological changes. Longitudinal monitoring of immune trajectories offers a more informative approach, enabling identification of deviations from normal immune dynamics prior to clinical manifestation (6, 22, 76).

### 8.6 Toward biomarker-guided pregnancy management

Despite these challenges, Treg-based immune biomarkers hold significant potential for advancing personalized obstetric care (6, 255). Integration of immune signatures with clinical, imaging, and biochemical data may enable more accurate prediction of pregnancy complications and improved risk stratification (256). Such approaches could facilitate earlier intervention and allow tailoring of monitoring and therapeutic strategies to individual patient profiles (27, 80). Ultimately, effective implementation will likely require composite models that integrate multiple dimensions of immune regulation rather than relying on single biomarkers. Continued advances in immune profiling technologies, coupled with large-scale longitudinal studies, will be critical for translating Treg biology into clinically actionable tools and for improving outcomes in pregnancy disorders (6, 119, 257).

**Table 2** summarizes representative Treg-related biomarkers across pregnancy disorders, highlighting how different dimensions of Treg biology particularly functional competence, spatial context, and immune balance contribute to diagnostic and prognostic utility.

**Table 2:** Treg biomarkers with diagnostic or predictive potential.

Biomarker	Sample	Disease	Clinical utility	References
FOXP3^+^ Treg frequency	Peripheral blood	Preeclampsia (PE)	Predictive	(124, 145)
FOXP3^+^CD25^+^ Treg proportion	Peripheral blood	Recurrent pregnancy loss (RPL)	Diagnostic	(240, 258)
Th17/Treg ratio	Peripheral blood	RPL, PE	Diagnostic/Predictive	(240, 259)
CD4^+^CD25^high^CD127^low/−^FOXP3^+^ Tregs	Peripheral blood	PE	Predictive	(20, 131)
Decidual FOXP3^+^ Treg density	Decidua/placenta	PE	Prognostic	(20, 58)
CTLA-4^+^ Treg expression	Peripheral blood	RPL	Diagnostic	(121, 260)
IL-10-producing Tregs	Peripheral blood	PTL	Predictive	(22, 27)
TIGIT^+^ Tregs	Peripheral blood	PE	Predictive	(261, 262)
Decidual Treg/uNK cell ratio	Decidua	PE, placental insufficiency	Prognostic	(14, 210)
Treg-associated chemokines (e.g., CCL22 recruitment axis)	Placenta/decidua	PE	Prognostic	(10, 263)

## 9 Therapeutic strategies to restore Treg-mediated immune tolerance

Given the central role of Tregs in maintaining maternal-fetal immune tolerance, therapeutic strategies aimed at restoring or enhancing Treg function have attracted increasing interest as potential approaches for managing pregnancy disorders (6, 264). Rather than broadly suppressing immunity, these strategies seek to re-establish regulatory balance by promoting Treg expansion, stabilizing suppressive function, or reshaping the immune microenvironment at the maternal-fetal interface (18, 28). Current approaches can be broadly categorized into cytokine-based modulation, immunosuppressive or immune-rebalancing agents, microbiome-targeted interventions, antigen-specific tolerance strategies, and emerging cell-based therapies (28).

### 9.1 Established clinical management and its relationship to immune tolerance

Although considerable attention has focused on emerging Treg-directed therapeutic strategies, current clinical management of pregnancy complications continues to rely primarily on established interventions such as low-dose aspirin, low-molecular-weight heparin (LMWH), corticosteroids, and hydroxychloroquine in selected patient populations (265–267). These therapies were not originally developed to target Treg biology directly; however, several exhibit immunomodulatory properties that may influence maternal-fetal immune tolerance.

Low-dose aspirin and LMWH are widely used in women at increased risk of preeclampsia, placental insufficiency, or recurrent pregnancy loss, primarily through antithrombotic and vascular mechanisms (268). Corticosteroids are occasionally employed in selected immune-mediated reproductive disorders because of their broad anti-inflammatory effects (269). Hydroxychloroquine has attracted increasing interest because of its ability to modulate innate immune activation, cytokine production, and antigen presentation. Emerging evidence further suggests that it may indirectly influence regulatory immune pathways involved in maternal-fetal tolerance (267, 270). While the precise effects of these therapies on Treg abundance and function remain incompletely defined, their established clinical use provides an important translational context for evaluating emerging Treg-targeted interventions. Future therapeutic strategies may therefore complement, rather than replace, existing standards of care by more specifically restoring immune tolerance mechanisms underlying pregnancy complications.

### 9.2 Cytokine-based modulation of Tregs

Cytokine signaling plays a central role in Treg differentiation, expansion, and lineage stability. Among these cytokines, interleukin-2 (IL-2) is particularly important for maintaining Treg survival and suppressive activity (52). Low-dose IL-2 therapy has been investigated in several immune-mediated diseases as a strategy to selectively expand Tregs without substantially activating effector T cells (52, 271). Although clinical data in pregnancy remain limited, preclinical findings suggest that enhancing IL-2 signaling may help restore immune tolerance in conditions characterized by Treg insufficiency (272).

Other anti-inflammatory cytokine pathways, including IL-10- and TGF-β-associated signaling, also contribute to maintenance of regulatory immune environments (12). Therapeutic strategies that reinforce these pathways may help stabilize Treg-mediated suppression and counteract inflammatory circuits implicated in pregnancy disorders (28, 273). However, because cytokine effects are highly context dependent, safety, dosage, and timing require careful evaluation in pregnancy.

### 9.3 Calcineurin inhibitors and immunosuppressive agents

Pharmacologic agents that suppress excessive effector T-cell activation have also been explored in selected pregnancy-related immune disorders (20, 22). Calcineurin inhibitors such as tacrolimus have been investigated in recurrent implantation failure and recurrent pregnancy loss associated with immune dysregulation (274). These agents do not directly expand Tregs, but by dampening inflammatory activation they may shift the immune balance toward restoration of regulatory control.

Clinical studies suggest that tacrolimus may improve outcomes in selected patients with immune-mediated reproductive failure, particularly those with abnormal Th1/Th2 immune ratios or heightened inflammatory responses (275, 276). By reducing pro-inflammatory signaling, calcineurin inhibition may indirectly support Treg recovery or functional dominance (52). Nevertheless, the use of systemic immunosuppressive agents in pregnancy requires careful risk-benefit assessment, given the need to preserve maternal host defense and fetal safety (277).

### 9.4 Microbiome-targeted interventions

As discussed in earlier sections, the maternal microbiome shapes immune homeostasis and Treg differentiation through microbial metabolites and immune signaling pathways (278). Microbiome-targeted interventions therefore represent an indirect but potentially important strategy for enhancing Treg-mediated immune tolerance (279). Probiotics, dietary interventions, and related approaches may alter the production of metabolites such as short-chain fatty acids, which promote Treg differentiation and stabilize FOXP3 expression (279). Although clinical evidence in pregnancy remains limited, emerging studies suggest that modulation of the gut microbiome may reduce systemic inflammatory signaling and improve immune balance (280). Further work is needed to determine whether microbiome-based strategies can reproducibly enhance Treg function and provide clinically meaningful benefit in pregnancy disorders (281, 282).

### 9.5 Antigen-specific immune tolerance approaches

A more precise therapeutic concept involves induction of antigen-specific tolerance toward fetal or paternal antigens. Unlike generalized immunosuppression, antigen-specific approaches aim to selectively suppress immune responses directed against fetal tissues while preserving broader immune competence (1, 9).

Conceptually, such strategies could include tolerogenic antigen-presenting cell-based immune modulation (for example, approaches designed to promote regulatory antigen presentation), selective induction or expansion of antigen-enriched regulatory T-cell populations, or peptide-guided immune tolerance strategies (6). However, these approaches remain highly experimental in reproductive immunology, and several major translational challenges remain unresolved. These include identification of clinically relevant paternal or fetal target alloantigens, uncertainty regarding whether tolerance induction would be most feasible through ex vivo cellular manipulation or *in vivo* immune modulation, optimal timing relative to pregnancy stage, and the critical need to avoid unintended systemic immunosuppression or interference with protective maternal immune responses (13, 50).

Experimental strategies include tolerogenic dendritic cells, peptide-based approaches, and immune checkpoint modulation designed to expand antigen-specific Tregs (283, 284). In principle, such approaches could provide a more selective means of restoring maternal-fetal tolerance with less risk of systemic immunosuppression (4, 9). Although these strategies remain largely experimental in reproductive immunology, advances in transplantation and autoimmunity suggest a plausible path toward pregnancy-specific tolerance therapies (21, 285).

### 9.6 Cell-based Treg therapies

Adoptive transfer of ex vivo expanded Tregs represents one of the most targeted emerging strategies for restoring immune tolerance (286). In this approach, Tregs are isolated, expanded under controlled conditions, and reinfused to enhance regulatory immune activity (12, 226). Clinical studies in transplantation and autoimmune diseases have demonstrated that adoptive Treg therapy can be feasible, safe, and capable of inducing durable tolerance in selected settings (286, 287).

Application in pregnancy disorders remains largely theoretical, but the concept is highly relevant for severe immune-mediated complications. In particular, the possibility of generating antigen-specific Tregs directed toward paternal or fetal antigens offers a compelling precision strategy for restoring maternal-fetal tolerance in selected patients (6, 9). However, a major unresolved limitation is the current lack of precise knowledge regarding the key paternal or fetal alloantigens recognized by maternal regulatory T cells that are required for maintenance of maternal-fetal tolerance, which substantially constrains rational antigen-directed therapeutic design (6, 9). Additional translational barriers include manufacturing complexity, uncertainty regarding optimal timing of administration, and the critical need to ensure maternal-fetal safety without disrupting protective immune competence (9, 13).

**Figure 4:**
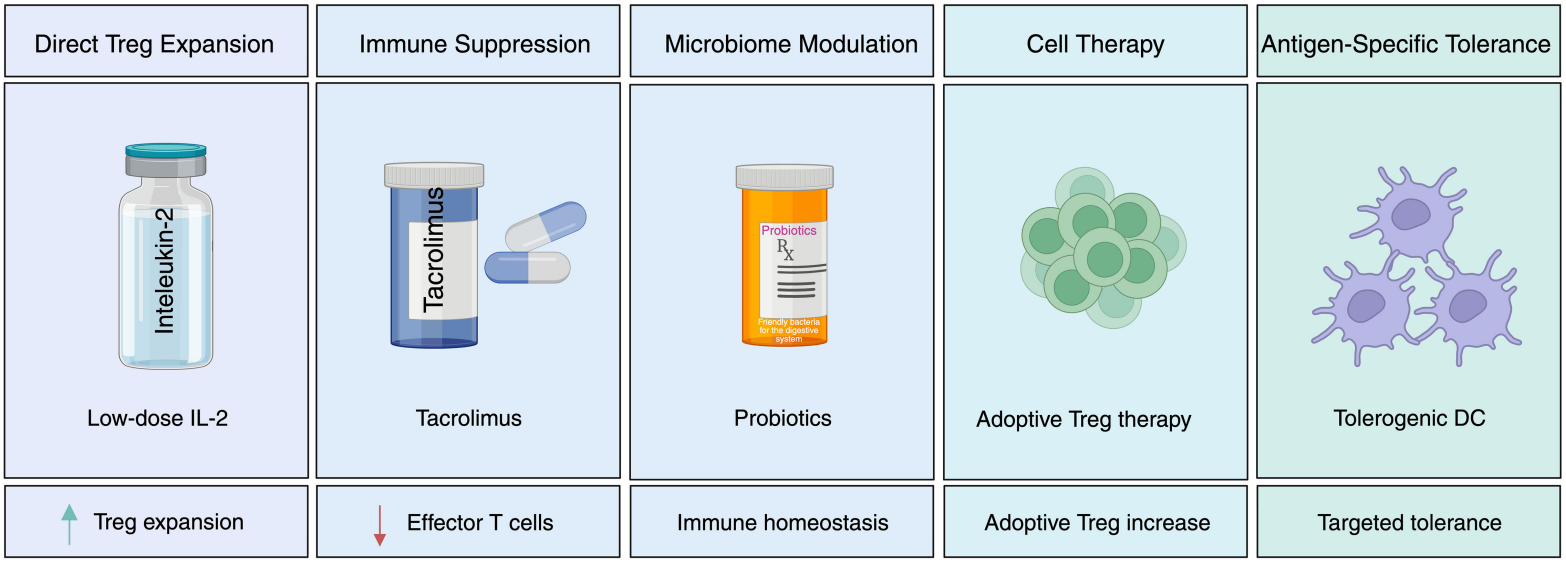
Therapeutic strategies targeting Treg pathways in pregnancy-related immune dysregulation. This schematic summarizes major therapeutic strategies aimed at restoring Treg-mediated immune tolerance in pregnancy-related disorders. Approaches are grouped by mechanism, including direct Treg expansion, indirect immune modulation, microbiome-targeted interventions, cell-based therapy, and conceptual antigen-directed tolerance strategies. Low-dose IL-2 promotes selective Treg expansion and may indirectly favor restoration of regulatory immune balance. Microbiome-targeted approaches, including probiotics and short-chain fatty acid-associated pathways, may influence Treg differentiation and immune homeostasis through metabolic and immunological signaling. Adoptive Treg therapy involves ex vivo expansion and reinfusion of Tregs to restore suppressive immune function. Antigen-directed tolerance approaches are presented as conceptual future strategies that may involve tolerogenic antigen-presenting cell–based immune modulation or selective enhancement of regulatory immune responses; however, these remain investigational in reproductive immunology due to unresolved challenges in antigen selection, implementation, timing, and maternal-fetal safety.

To contextualize these emerging therapeutic approaches, **Figure 4** summarizes major strategies targeting Treg pathways according to their mechanisms and downstream immunological effects.

### 9.7 Safety considerations in pregnancy

Despite the promise of Treg-targeted therapies, safety remains a central concern in the development of immunomodulatory interventions during pregnancy (120). Any therapy that alters maternal immune responses must strike a careful balance between restoring tolerance and preserving effective host defense against infection. Potential effects on fetal development must also be considered (21, 288). For this reason, therapeutic strategies should aim to restore physiological immune balance rather than induce broad immunosuppression. Biomarker-guided patient stratification, precision dosing, and mechanism-informed therapeutic selection may help identify those patients most likely to benefit while minimizing maternal and fetal risk (289). From a translational perspective, the future of this field will likely depend on tailoring intervention to the dominant dimension of Treg dysfunction whether quantitative insufficiency, functional impairment, lineage instability, or disturbed immune crosstalk.

To provide a translational overview, **Table 3** summarizes representative clinical and preclinical studies targeting Treg-related pathways in pregnancy disorders, highlighting therapeutic strategies, study design, and immunomodulatory effects.

**Table 3:** Representative clinical and preclinical studies targeting Treg-related pathways in pregnancy disorders.

Therapy	Disease	Study type	Outcome	References
Tacrolimus	RPL, recurrent implantation failure	Clinical study	Improved pregnancy rate and reduced inflammatory T-cell responses	(276, 290)
Low-dose IL-2	Autoimmune disease models; immune dysregulation	Preclinical/early clinical	Selective expansion of Tregs	(273, 291)
Progesterone therapy	Recurrent miscarriage risk	Clinical study	Immunomodulatory effects with Treg expansion	(64, 292)
Intravenous immunoglobulin (IVIG)	Recurrent pregnancy loss	Clinical study	Immune modulation and increased Treg function	(293, 294)
Vitamin D supplementation	Recurrent miscarriage/infertility	Observational/clinical	Increased FOXP3 expression and Treg differentiation	(295, 296)
Probiotics/microbiome modulation	Preeclampsia risk	Observational studies	Immune modulation and improved inflammatory balance	(297, 298)
Tolerogenic dendritic cells	Pregnancy tolerance models	Preclinical	Induction of antigen-specific Tregs	(299, 300)
Adoptive Treg cell therapy	Autoimmune and transplantation models	Preclinical/early clinical	Restoration of immune tolerance via expanded Tregs	(212, 301)
mTOR pathway modulation (e.g., rapamycin)	Autoimmune disease models	Preclinical	Promotes Treg differentiation and stability	(302, 303)

Collectively, these studies support the therapeutic relevance of targeting Treg pathways, while also underscoring that most current approaches remain early in translational development and require more robust, well-controlled evaluation of efficacy, timing, and safety in pregnancy.

## 10 Key unresolved questions and future directions

Despite substantial progress in defining the role of Tregs in pregnancy, several fundamental questions remain unresolved. Although current evidence clearly supports the importance of Tregs in maintaining maternal-fetal immune tolerance, important gaps persist in understanding the causal mechanisms underlying Treg dysfunction, identifying the biologically relevant Treg populations responsible for maternal-fetal immune tolerance, defining the relationship between circulating and tissue-resident Tregs, and translating these insights into clinically actionable biomarkers and therapeutic strategies (28, 64). Rather than revisiting individual observations discussed throughout this review, the following sections prioritize six key research areas that are likely to have the greatest impact on advancing the field. Addressing these challenges will require a shift from descriptive characterization toward integrative, multi-dimensional approaches that combine mechanistic immunology, spatially resolved and single-cell technologies, systems-level analysis, and longitudinal clinical investigation. In particular, future research should focus on how distinct dimensions of Treg biology including abundance, functional competence, lineage stability, antigen specificity, and spatial organization interact across gestation to influence pregnancy outcomes and therapeutic responsiveness.

### 10.1 Causality versus association

A central challenge in reproductive immunology is determining whether Treg alterations represent primary drivers of pregnancy complications or secondary consequences of underlying pathology (9, 28). Although numerous studies report associations between reduced Treg abundance or impaired function and conditions such as recurrent pregnancy loss, preeclampsia, and preterm labor, causal relationships remain difficult to establish within the complex immune environment of the maternal-fetal interface (1, 64). Resolving this question will require longitudinal and mechanistic studies that define the temporal sequence of immune dysregulation. Tracking immune trajectories from preconception through gestation may help determine whether Treg dysfunction precedes clinical disease or arises as a downstream effect. Such approaches could also identify critical windows during which therapeutic intervention may restore immune balance and prevent disease progression.

### 10.2 Antigen specificity of pregnancy-associated Tregs

The antigen specificity of Tregs involved in maternal-fetal tolerance remains incompletely understood. Although experimental evidence suggests expansion of paternal antigen-specific Tregs during pregnancy, the extent to which antigen-specific recognition drives immune tolerance in humans is still unclear (1, 9). Defining antigen specificity is particularly important for advancing precision immunotherapy. Identification of fetal or paternal antigen-specific Tregs could enable targeted approaches that restore tolerance without broad immunosuppression. Advances in T-cell receptor sequencing, single-cell profiling, and lineage tracing technologies offer promising tools for dissecting antigen-specific Treg responses in both decidual and systemic compartments (9, 210).

### 10.3 Maternal-fetal compartmentalization of immune responses

The maternal-fetal interface represents a spatially specialized immune environment in which local immune regulation may diverge significantly from systemic immune activity (40). Decidual Tregs exhibit distinct phenotypes and functional properties shaped by local microenvironmental cues, yet the relationship between circulating and tissue-resident Treg populations remains poorly defined (14). Future research should focus on how immune regulation is coordinated across these compartments, including the mechanisms governing Treg trafficking, retention, and functional adaptation within placental tissues. Elucidating these spatial dynamics will be essential for interpreting immune biomarkers and for designing therapies that effectively target local immune regulation (1, 64).

### 10.4 Personalized immune stratification

Pregnancy disorders such as preeclampsia and recurrent pregnancy loss are highly heterogeneous, with diverse underlying mechanisms that may or may not involve immune dysregulation (304, 305). Consequently, a major challenge is identifying the subset of patients in whom Treg dysfunction represents a dominant pathogenic driver. Integration of immune profiling with clinical, genetic, and environmental data may enable development of stratification models that distinguish immune-mediated from non-immune disease pathways. Such models could guide patient selection for immunomodulatory therapies and improve the precision of clinical decision-making (28).

### 10.5 Feasibility of precision immunotherapy in obstetrics

Although advances in immunotherapy have transformed the management of many immune-mediated diseases, their application in obstetric medicine remains limited. A key challenge lies in developing strategies that restore physiological immune balance without compromising maternal host defense or fetal safety (21). Future therapeutic approaches should prioritize targeted modulation of immune pathways based on underlying mechanisms of Treg dysfunction. Biomarker-guided interventions, antigen-specific tolerance strategies, and cell-based therapies may offer opportunities for precision immunotherapy, but their clinical implementation will require rigorous evaluation of safety, timing, and efficacy in the maternal-fetal context (306, 307).

### 10.6 Integrating multi-omics and systems immunology

The rapid development of single-cell sequencing, spatial transcriptomics, and multi-omics technologies provides unprecedented opportunities to characterize immune regulation during pregnancy at high resolution (308). Integration of these datasets within systems immunology frameworks may reveal emergent properties of immune networks that cannot be captured by single-parameter analyses (1).

Large-scale, collaborative studies combining multi-omics profiling with longitudinal clinical data will be essential for translating these insights into practice. Such approaches may enable identification of predictive immune signatures, elucidation of disease mechanisms, and development of personalized therapeutic strategies that reflect the multi-dimensional nature of Treg biology within broader maternal–fetal immune networks (309, 310).

Importantly, dysregulation of pregnancy immune tolerance may not arise solely from intrinsic Treg defects but may also reflect alterations in interconnected immune populations that shape regulatory function, including B cells, dendritic cells, macrophages, NK cells, and other stromal or immune compartments (4, 311). For example, impaired expansion of regulatory immune populations such as IL-10-producing B cells could indirectly compromise Treg maintenance and broader maternal-fetal immune tolerance (45, 312).

Together, these unresolved questions highlight the need for a unified framework that integrates temporal, spatial, and functional dimensions of Treg biology. Addressing these challenges will be critical for translating mechanistic insights into clinically actionable strategies that improve pregnancy outcomes.

## 11 Conclusion

Pregnancy represents a unique immunological state in which the maternal immune system must simultaneously maintain tolerance toward the semi-allogeneic fetus while preserving protective immunity against pathogens. Tregs have emerged as central regulators of this balance, coordinating immune suppression, tissue remodeling, and vascular adaptation at the maternal-fetal interface.

A key insight from recent studies is that Treg dysregulation in pregnancy disorders cannot be explained solely by changes in cell abundance. Instead, pathological states reflect multi-dimensional alterations in Treg biology, including impaired suppressive function, reduced lineage stability, altered spatial distribution, and disruption of regulatory signaling networks. These interconnected dimensions collectively determine the integrity of immune tolerance during pregnancy.

Advances in high-dimensional immune profiling including single-cell, spatial, and multi-omics approaches are redefining Treg biology as a dynamic, heterogeneous, and context-dependent system. These technologies are beginning to uncover how regulatory immune networks evolve across gestation and how their disruption contributes to pregnancy complications. Importantly, they provide a foundation for developing multi-dimensional biomarkers that move beyond static measurements toward functional and spatially resolved immune assessment.

Despite these advances, significant barriers remain to clinical translation. Key challenges include establishing causal relationships between Treg dysfunction and disease, standardizing immune profiling methodologies, and integrating systemic and tissue-specific immune information. Addressing these gaps will require longitudinal, multi-center studies that combine mechanistic investigation with robust clinical validation.

Looking forward, the integration of mechanistic immunology with biomarker development and therapeutic innovation may enable a new generation of precision approaches in obstetric medicine. Biomarker-guided strategies incorporating Treg-related immune signatures, together with emerging therapies aimed at restoring regulatory balance, hold promise for improving early detection, risk stratification, and treatment of pregnancy complications.

In summary, Tregs represent a central nexus linking immune tolerance, placental biology, and maternal health. A framework that integrates quantitative, functional, spatial, and temporal dimensions of Treg biology will be essential for translating these insights into clinically actionable strategies and advancing the management of pregnancy disorders.
